# Effective Dose Evaluation of Cerebral Angiography Using Kerma Area Product and Development of Effective Dose Conversion Factors

**DOI:** 10.3390/diagnostics16152467

**Published:** 2026-08-05

**Authors:** Jeongbok Min, Jungsu Kim

**Affiliations:** 1 Department of Bio-Health Convergence Major in Medical Convergence Radiologic-Technology, Graduate School of Health Technology, Daegu Health College, Daegu 41453, Republic of Korea; jb1968@ulsanjoongang.co.kr; 2 Department of Radiology, Ulsan Joongang Hospital, Ulsan 44667, Republic of Korea; 3 Department of Radiological-Technology, Daegu Health College, Daegu 41453, Republic of Korea

**Keywords:** transfemoral cerebral angiography, Kerma area product, effective dose, conversion factor, risk assessment

## Abstract

**Background/Objectives**: Transfemoral cerebral angiography (TFCA) involves significant radiation exposure because of prolonged fluoroscopy and repeated imaging sequences. Because complex dose simulations are often infeasible in routine clinical practice, this study employed PC-based Monte Carlo methods to estimate organ-absorbed and effective doses. The primary objective was to establish Kerma area product (KAP)-based conversion factors to facilitate efficient clinical dose assessment. **Methods**: We analyzed 30 patients who underwent TFCA. Key parameters, including total KAP, tube voltage, and projection angles, were obtained from DICOM Radiation Dose Structured Reports. Monte Carlo simulations were conducted for anteroposterior, lateral, and cranial planes. The conversion factors were then derived by calculating the ratio of the effective dose to the total KAP. **Results**: The average KAP was 36.23 ± 6.81 Gy·cm^2^. Effective doses calculated via Monte Carlo simulation were 1.70 ± 0.35 mSv under International Commission on Radiological Protection (ICRP) 60 and 1.90 ± 0.40 mSv under ICRP 103 standards. The corresponding conversion factors were 0.0468 ± 0.0054 and 0.0525 ± 0.0065 mSv/Gy·cm^2^ respectively. Notably, the ICRP 103-based factor was 12.2 percent higher than the ICRP 60-based factor, reflecting updated tissue weighting factors in modern dosimetry. **Conclusions**: This study provides standardized conversion factors that allow rapid and reliable dose estimation without requiring complex simulations. The findings demonstrated substantial radiation absorption in the head and neck regions, providing critical data for routine dose monitoring. These results emphasize the importance of rigorous radiation protection and safety protocols during interventional procedures.

## 1. Introduction

In modern clinical practice, advances in imaging technology and the increasing use of interventional procedures for diagnosis and treatment have contributed to a steady rise in medical radiation exposure [1,2]. As the use of X-rays for diagnosis and treatment has become increasingly common in radiology, the population radiation dose attributable to medical imaging has also continued to rise [2], and accordingly, the importance of medical radiation management is being further emphasized [3]. Medical radiation can cause biological changes at molecular and cellular levels by transferring energy within human tissues, and the extent of these effects depends on the absorbed dose and the properties of the exposed organs [3,4,5]. The biological effects of radiation exposure are broadly categorized into stochastic and deterministic effects. Stochastic effects have no threshold dose, and the likelihood of long-term complications, such as cancer, increases with radiation exposure [3,4]. On the other hand, deterministic effects are reported to manifest as tissue reactions such as skin damage, erythema, hair loss, and cataracts above a specific threshold dose [3,4,5]. To address these radiation risks, the International Commission on Radiological Protection (ICRP) recommends the justification and optimization of medical radiation use through adherence to the “as low as reasonably achievable” principle [3,5]. The concept introduced to quantitatively evaluate radiation hazards is the effective dose. The effective dose is a protection quantity calculated by employing tissue weighting factors (WT) to the organ absorbed dose, and it is utilized to integrally evaluate the radiation hazard to the entire human body [3,6]. ICRP Publication 103 reorganized the tissue weighting factors by reflecting the latest epidemiological and radiobiological research results, and it is currently widely utilized as the international standard for effective dose evaluation in the field of medical imaging. Transfemoral Cerebral Angiography (TFCA) is a key diagnostic and interventional imaging technique capable of visualizing complex cerebrovascular structures in high resolution, and its clinical utility is extremely high [7,8]. Since TFCA involves radiation-sensitive organs such as the eyes within the examination area, requires long-term fluoroscopy, and includes repeated image acquisition with multiple projection angle adjustments for lesion assessment, patients may receive a considerable radiation dose during the procedure [9]. These features result in higher absorbed doses to head and neck organs, including the eyes and thyroid, which have been associated with an increased risk of both stochastic and deterministic effects [10]. In routine clinical settings, clinicians who are not trained in health physics often find it difficult to conduct specialized simulations to estimate effective dose. Although previous studies have evaluated effective doses using physical phantoms, these approaches are limited to simulated conditions and do not reflect real-world patient data [11]. To address this limitation, the present study utilizes baseline data from actual Korean patients to evaluate the effective dose of transfemoral cerebral angiography (TFCA) in a clinical environment. Specifically, this study aims to quantitatively assess organ-absorbed and effective doses through PC-based Monte Carlo simulations using real TFCA clinical data. Furthermore, we aim to develop effective dose conversion factors based on the Kerma-Area Product (KAP) to provide medical staff with a practical tool for easily calculating patient doses in clinical settings.

## 2. Materials and Methods

### 2.1. Study Design and Patients

In this study, an evaluation of effective dose was conducted using a PC-based Monte Carlo simulation, PCXMC 2.0 (Finnish Radiation and Nuclear Safety Authority [STUK], Finland) on the digital image communications in medicine (DICOM) radiation dose structured reports (RDSR) of 30 adult patients (15 males and 15 females) who underwent TFCA at a medical institution in Ulsan, Korea, from November 2023 to November 2025. The equal gender distribution was intentionally established to mitigate gender-related dosimetric bias. To minimize inter-operator variability, all diagnostic TFCA procedures included in this study were performed by a single board-certified neurosurgeon with 5 years of dedicated experience specifically in performing TFCA. Patients were excluded if they required additional radiographic projections beyond the standard anteroposterior, lateral, and cranial views. Furthermore, to control for variations arising from differences in patient physique, inclusion was strictly limited to individuals weighing between 55.2 kg and 73.95 kg. This range corresponds to the 25th and 75th percentiles of the 8th Size Korea standard anthropometric survey [12]. Because these rigorous criteria were applied retrospectively as predefined database filters, eligible patients were consecutively selected until the target sample size of 30 was achieved. Consequently, patients falling outside the weight range were implicitly excluded during the extraction process, and their exact numbers were not formally tracked. The retrospective study protocol was approved by the institutional review board of our institution (IRB No. DHCIRB-2026-03-0001), which waived the requirement for informed consent.

### 2.2. Imaging Equipment and Simulation

To perform the PC-based Monte Carlo simulation, information was collected regarding the total KAP, tube voltage (kVp), filter thickness, field size, detector source distance, projection angles, imaging modes (fluoroscopy and DSA), and characteristics of the angiographic equipment for each examination. Data for the simulation were gathered based on angiographic equipment records and the RDSR provided by the picture archiving and communication system (PACS). Patient characteristic data consisted of sex, age, height, and weight, while dose-related items secured the KAP values recorded in both fluoroscopy and X-ray image acquisition modes. All examinations were performed using a biplane DSA system (Alphenix Biplane BLA-900A; Canon Medical Systems, Otawara, Japan). The imaging protocol for TFCA was optimized as follows: neuro-DSA mode was set at 3 frames/s with 80 kVp, utilizing a 0.3 mm Cu filter and a 0.4 mm focal spot size. Fluoroscopy was operated in pulse mode at 15 pulses/s with 70 kVp and a 0.5 mm Cu filter. Both imaging modes employed wedge-type strip filters to improve image quality while minimizing the radiation dose.

The KAP was monitored using an integrated transmission ionization chamber (VacuTec Messtechnik GmbH in Dresden, Germany), which is embedded within the collimator housing of the Alphenix Biplane system. The integrated dose monitoring system underwent annual calibration conducted by a certified medical physicist in collaboration with a Canon Medical Systems service engineer. This process ensured that the measurement accuracy remained within the ±35% tolerance range specified by IEC 60601-2-43 standards. Calibration was conducted using a reference ionization chamber traceable to national standards, thereby confirming the reliability of the dose data recorded in the RDSR for subsequent simulations [13].

The simulation was performed using the Adult Cristy–Eckerman mathematical phantom for each irradiation angle, based on parameters obtained from the RDSR. X-ray irradiation geometry information for the TFCA examination was entered independently for the anteroposterior (AP), lateral, and cranio-caudal angles projection planes. X-ray irradiation conditions were simulated using the actual field size, DSD and tube voltage used during the examinations for the AP direction (0°), lateral direction (90°), and CCA ranging from cranial 20° to 30°.

The exposure angle and collimator field size for the fluoroscopy and acquisition modes were extracted from the DICOM RDSR irradiation event data and consistently applied to the PCXMC simulation. For each procedure, the simulation phantom was specifically customized based on the height, weight, sex, and age of the individual patient, and the number of photon histories was set to 2 × 10^5^ per calculation.

### 2.3. Conversion Factor Calculations

In this study, the KAP-based effective dose conversion factor (K) was calculated using the patient-specific effective dose (E, mSv) derived through the PCXMC 2.0 Monte Carlo simulation and the KAP (Gy·cm^2^) values recorded by the angiographic equipment. The formula for the effective dose conversion factor relative to the KAP is shown in Equation (1).

(1) $$K=\frac{E}{KAP}$$

K = Conversion factor, E = effective dose (mSv), KAP = kerma area product (Gy·cm^2^).

### 2.4. Statistical Analysis

All statistical analyses were performed using IBM SPSS Statistics version 22 (IBM Corp., Armonk, NY, USA). Continuous variables are expressed as means ± standard deviations (SD). To justify the use of parametric tests, the normality of the data distribution for all continuous variables—including KAP, effective doses (ICRP 60 and ICRP 103), and conversion factors (K60 and K103)—was evaluated using the Shapiro–Wilk test. Because all analyzed variables exhibited *p*-values greater than 0.05, the assumption of normality was met. Consequently, an independent samples *t*-test was utilized to compare the mean differences between the male and female groups.

To validate the derived conversion factors, an internal validation was conducted using the leave-one-out cross-validation (LOOCV) approach due to the small sample size (*N* = 30). For each iteration, one patient was excluded to calculate the conversion factor from the remaining 29 patients, which was then used to estimate the dose for the excluded patient. The prediction performance of this validation was quantified using the mean absolute error (MAE) and mean absolute percentage error (MAPE), while overall reliability and correlation were determined via linear regression analysis (R^2^). A *p*-value of <0.05 was considered statistically significant.

## 3. Results

### 3.1. Effective Dose and Absorbed Dose

A total of 30 patients were included, with an equal sex distribution of 15 males and 15 females. Regarding the demographic characteristics of the study subjects, the average age was 56.3 years for males and 61.5 years for females. The average weight was 64.5 ± 9.0 kg, and the average height was 164.9 ± 8.6 cm.

For all target patients, the average KAP of X-rays irradiated during the TFCA examination was 36.23 ± 6.81 Gy·cm^2^. The average effective dose evaluated through Monte Carlo simulation was 1.70 ± 0.35 mSv based on ICRP 60 factors, and 1.90 ± 0.40 mSv based on ICRP 103 factors, as shown in Table 1.

As a result of comparing the radiation dose by sex, the mean value of the KAP was 37.98 Gy·cm^2^ for males and 34.48 Gy·cm^2^ for females, indicating slightly higher KAP values in the male patient group. Conversely, the effective doses derived through simulation exhibited a different trend depending on sex.

The mean effective doses by sex were 1.65 mSv on ICRP 60 and 1.84 mSv on ICRP 103 for males, while for females, they were 1.74 mSv on ICRP 60 and 1.97 mSv on ICRP 103. Although the KAP values were lower in females than in males, the effective doses were higher in females. In Table 2, the respective effective doses and KAP values are presented. In summary, although the KAP was higher in males, the calculated effective dose was greater in females.

The mean KAP for males was 37.98 ± 7.69 Gy·cm^2^, which was higher than the 34.48 ± 5.52 Gy·cm^2^ observed in females, yet this did not reach statistical significance. However, the ICRP 103 conversion factor of 0.057 ± 0.006 in females was significantly greater than the 0.048 ± 0.004 recorded in males, as evidenced by a *t*-statistic of -4.759 with 28 degrees of freedom and a *p*-value of less than 0.001, resulting in an overall higher mean effective dose in the female group.

Analysis of the absorbed dose by organ, calculated through simulation, demonstrated that the upper spine had the highest absorbed dose among all subjects, followed by the skull, scapula, clavicle, and humerus. The results are illustrated in Table 3. These organs were all confirmed to be anatomical structures located within the direct irradiation area or adjacent to the field of view during TFCA examinations.

When stratified by sex, the group of organs receiving the highest absorbed doses were similar in both males and females. However, in certain organs such as the scapula and humerus, sex-based differences were noted, with females showing a higher mean absorbed dose than males, as presented in Table 3. The average absorbed dose for the scapula was 21.26 ± 5.88 mGy for males and 23.77 ± 5.59 mGy for females, while for the humerus, it was 12.73 ± 3.13 mGy for males and 14.23 ± 2.26 mGy for females.

As observed in the results shown in Table 3, radiation-sensitive organs located in the head and neck, such as the brain and thyroid, also revealed high absorbed doses, with overall averages of 8.76 ± 1.57 mGy and 8.48 ± 1.51 mGy, respectively. Conversely, organs in the abdominal and pelvic regions had low absorbed doses, some registering values of 0.01 mGy or less.

### 3.2. Effective Dose Conversion Factor

The effective dose conversion factor (K) was derived from the KAP by relating effective doses obtained from the PCXMC 2.0 simulation to the actual KAP values recorded by the equipment. As shown in Table 4, the analysis of all patients demonstrated that the conversion factor based on ICRP 60 was 0.0468 ± 0.0054 mSv/Gy·cm^2^, while the conversion factor based on ICRP 103 was 0.0525 ± 0.0065 mSv/Gy·cm^2^. The ICRP 103 conversion factor increased by approximately 12.2% compared to ICRP 60. This confirms that the ICRP 103 system, which reflects updated tissue weighting factors, yields a higher effective dose even under the same irradiation conditions.

**Figure 1:**
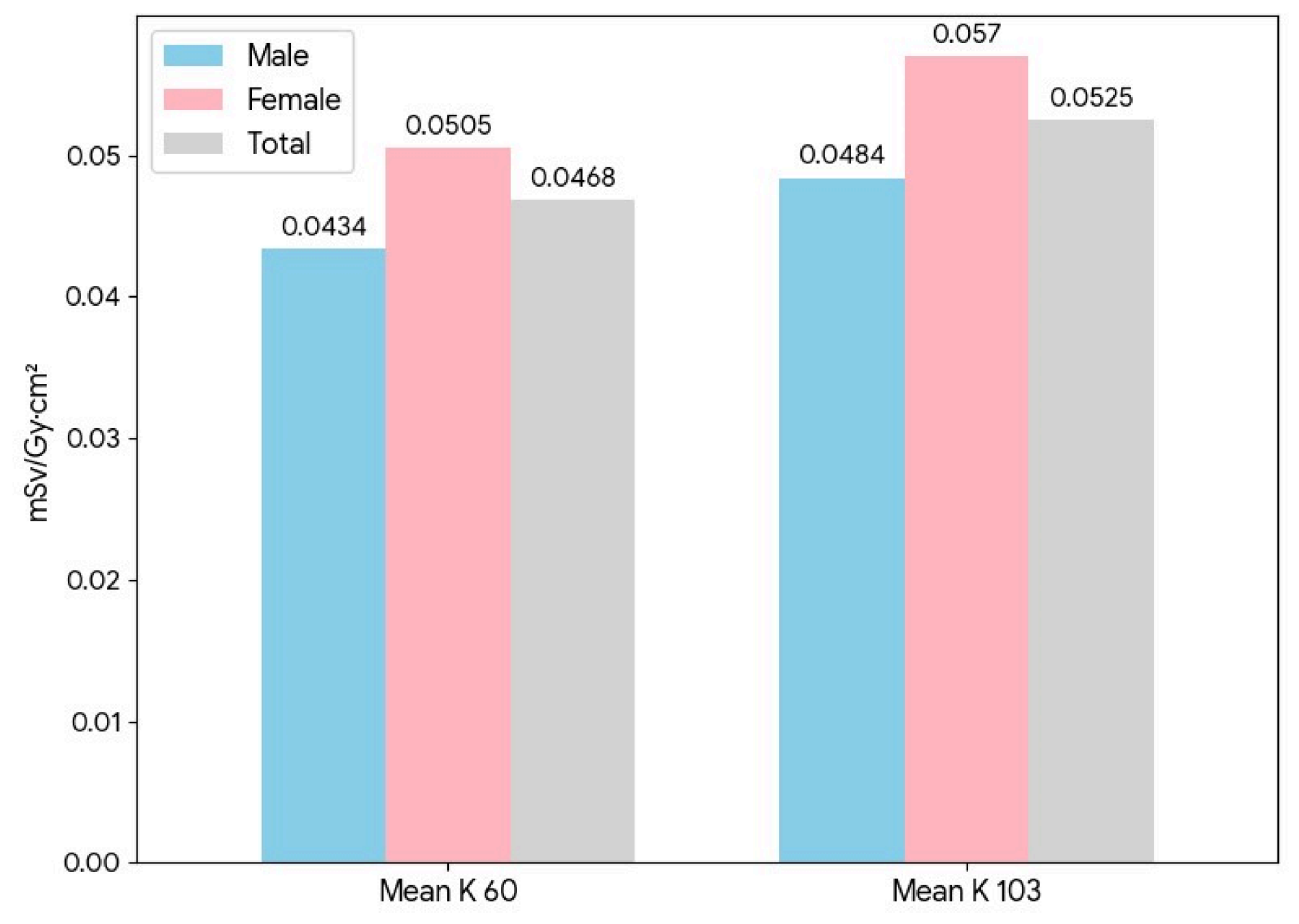
Sex-Specific Conversion Factors (K) Based on ICRP 60 and ICRP 103.

According to the analysis of conversion factors by sex, female patients demonstrated 0.0505 mSv/Gy·cm^2^ for ICRP 60 and 0.0570 mSv/Gy·cm^2^ for ICRP 103. Conversely, male patients demonstrated 0.0434 mSv/Gy·cm^2^ and 0.0484 mSv/Gy·cm^2^, respectively. The average conversion factors by sex presented in Figure 1. Across all standards, the conversion factors were higher in females than in males, indicating that females receive a higher estimated effective dose even under identical KAP conditions.

The LOOCV analysis demonstrated high accuracy for the derived conversion factor. The mean absolute error between the actual and estimated ICRP 103 values was 0.00035 ± 0.00028 mSv, with a mean absolute percentage error of 0.67%. The high correlation (R^2^ = 0.989) confirms the reliability of the conversion factor across the cohort.

### 3.3. Risk Assessment

The radiation associated risk of exposure induced death (REID) and loss of life expectancy (LLE) calculated using the risk assessment module of PC-based simulations. After analyzing a total of 30 patients, the average REID was 0.0070 ± 0.0028%, and the average loss of life expectancy was 10.56 ± 5.88 h. These results are summarized in Table 5. In the sex-specific analysis, the average REID was 0.0088 ± 0.0028% for female patients and 0.0053 ± 0.0012% for male patients, demonstrating higher values in females. Similarly, the loss of life expectancy was an average of 13.6 ± 6.4 h for females and 7.5 ± 3.3 h for males. In all risk indices, female patients exhibited higher figures than male patients.

## 4. Discussion

In this study, patient radiation exposure during diagnostic TFCA was quantitatively evaluated by calculating effective dose and organ-absorbed doses using the PCXMC 2.0 Monte Carlo simulation. By utilizing clinical data from 30 patients, this study incorporated specific irradiation conditions, physical characteristics, and exposure geometries for each case. Thus, the average effective dose based on ICRP Publication 103 was 1.90 mSv, and the average KAP was 36.23 Gy·cm^2^. In the study by Feygelman et al., the average effective dose per diagnostic TFCA was presented as 10.6 mSv according to ICRP60 standards; however, this is approximately 5.6-fold higher than the average effective dose of 1.90 mSv calculated in this study [14]. This difference suggests that patient radiation exposure levels were relatively higher in the past, when single-plane angiography systems were predominantly used and radiation reduction technologies were less widely implemented.

In a previous study by Yi et al. using a bi-plane angiography device, the average KAP was 232.12 Gy·cm^2^ and 148.54 Gy·cm^2^ depending on the device parameters [15]. This study indicated that the patient dose could be reduced depending on the device parameter settings. This is approximately 6.4-fold and 4.1-fold higher, respectively, than the average KAP of 36.23 Gy·cm^2^ in this study. Furthermore, this value is significantly lower than the KAP of 78.0 ± 43 Gy·cm^2^ reported in a previous study on diagnostic angiography conducted in a Korean population [16]. These results quantitatively demonstrate that patient dose in neuro-angiography can fluctuate significantly depending on the equipment generation, exposure parameter, examination protocol, operator proficiency, and the complexity of the procedure.

Analysis of organ-absorbed doses demonstrated increased exposure in the head, neck, and upper thoracic regions, particularly the skull, upper cervical spine, scapula, and clavicle. This dose distribution reflects the procedural nature of diagnostic TFCA, where the irradiation field is centered on the cranio–cervical axis, involving concentrated fluoroscopy and multi-angle acquisitions. In this study, the TFCA protocol utilized AP projections at 0 degrees, lateral projections at 90°, and cranial CCA projections ranging from 20 to 30°. Owing to their immediate proximity to the primary X-ray beam, the skull and scapula were subject to higher radiation loads.

In the AP projection, the under-tube configuration with the X-ray tube positioned posteriorly resulted in a higher dose to the upper spinal region. However, this geometric configuration inherently offers a protective effect by decreasing radiation exposure to radiosensitive organs in the anterior cranium. Furthermore, this setup facilitates closer proximity between the detector and the subject, which effectively suppresses scatter radiation and subsequently minimizes the radiation burden on the operator. These findings are consistent with previous studies investigating organ dose distribution during neuro-angiographic procedures.

Particularly, the skull and structures adjacent to the cervical spine are repeatedly exposed to direct primary radiation along the X-ray irradiation path and, accordingly, high absorbed doses can accumulate in the bone tissue and adjacent soft tissues. These characteristics can lead to a significant radiation exposure to radiation-sensitive head and neck organs, such as the lens of the eye, thyroid, and salivary glands, indicating that the radiation risk to these organs may increase. Thus, in high-dose interventional radiation procedures such as TFCA, precise dose evaluation considering the distribution of organ-absorbed doses is warranted in addition to effective dose-centered evaluation, and this serves as an important basis for establishing strategies to optimize patient radiation protection [17].

In the sex-based analysis, female patients exhibited higher effective dose, conversion factor, RIED, and LLE values than male patients. This finding likely reflects differences in body habitus influencing radiation absorption, along with the impact of higher tissue weighting factors assigned to female reproductive organs and breasts in ICRP Publication 103. These results indicate that a higher radiation risk is calculated for female patients even under the same KAP conditions, making a sex-aware radiation protection strategy clinically essential [18]. Furthermore, this study suggests that sex is an important consideration in dose estimation for neurointerventional procedures, as the markedly higher conversion factors and RIED/LLE values observed in females highlight the need for sex-specific factors to achieve accurate dose monitoring.

Comparative analysis of organ-absorbed doses revealed distinct sex-specific patterns between male and female cohorts. While male patients exhibited higher doses in skeletal structures such as the upper spine at 30.16 mGy and the skull at 24.44 mGy, female patients showed elevated doses in radiosensitive soft tissues, including the lungs at 4.01 mGy and the breasts at 1.24 mGy.

Despite higher individual organ doses in certain male skeletal regions, the average total body dose was higher in females at 3.86 mGy compared to 3.45 mGy in males. This discrepancy underscores the substantial impact of sex-based anatomical differences and the greater tissue weighting factors assigned to female organs in effective dose calculations, particularly under the ICRP 103 guidelines.

Specifically, during TFCA procedures, although the primary X-ray beam is confined to the cranial region, secondary scatter radiation inevitably exposes adjacent radiosensitive organs. Due to inherent geometric differences between the sexes, the spatial relationship and distance of these organs, particularly the breasts and the thyroid from the primary beam differ significantly, fundamentally altering the scatter absorption profile.

Furthermore, the ICRP 103 framework amplifies the impact of these differences by assigning an elevated tissue weighting factor to breast tissue. The combination of the relative proximity of female breast tissue to the cranial scatter field and its high weighting factor systematically elevates the calculated effective dose for women. Consequently, relying on a generic, sex-neutral conversion factor fails to capture these complex dosimetric dynamics and risks the miscalculation of patient doses. Therefore, adopting sex-specific conversion factors is logically and clinically essential for providing precise, individualized radiation risk assessments in routine practice.

A limitation of the PCXMC software used in this study is that it does not provide a discrete absorbed dose for the lens of the eye as a separate organ. However, it is noteworthy that the previous literature on diagnostic cerebral angiography has reported a mean lens dose of approximately 155 mGy [19]. Given that the lens is one of the most radiosensitive tissues, this reported value suggests that further investigations using specialized phantoms or direct measurements are warranted to precisely evaluate the ocular radiation risk during TFCA procedures.

Another key finding of this study is the derivation of a KAP-based effective dose conversion factor for use in diagnostic TFCA. The conversion factor calculated in this study was 0.053 mSv/Gy·cm^2^ on average based on ICRP Publication 103, which is highly significant in that it is an empirically based value derived through Monte Carlo simulation reflecting the latest biplane angiography equipment and actual clinical irradiation conditions. The conversion factor obtained in this study is significantly lower than the 0.09 mSv/Gy·cm^2^ derived in a previous study by Sanchez et al. [20]. The KAP is an index automatically recorded by angiography equipment; if the conversion factor presented in this study is employed, the patient’s effective dose can be estimated quickly and consistently immediately after the procedure without a separate complex calculation process. This provides practical benefits for patient dose monitoring, inter-examination comparisons, diagnostic reference level management, and the advancement of radiation protection education and quality assurance in clinical practice.

However, the conversion factor derived in this study represents an average radiation risk evaluation index based on a specific equipment configuration and examination protocol, and it does not fully reflect the differences in organ dose distribution according to individual patients’ body types, changes in irradiation angles, fluoroscopy time, and lesion complexity. Hence, when precise organ-absorbed dose evaluation or radiation risk analysis at the individual patient level is required, a detailed dose evaluation through Monte Carlo-based simulation, such as the one applied in this study, is warranted.

Since all examinations in this study were conducted by the same skilled operator, the effect of dose variability due to technical differences between operators was sufficiently controlled. In actual clinical practice, fluoroscopy time and the number of imaging acquisitions may vary depending on the difference in vascular anatomical structures per patient, especially the shape of the aortic arch, internal carotid artery access route, degree of vascular tortuosity, and complexity of the lesion; hence, the possibility that these factors influenced the variation in patient dose cannot be excluded.

Nevertheless, this study is noteworthy in that it presented the effective dose, organ-absorbed dose, and KAP-based effective dose conversion factors derived from Monte Carlo simulations that incorporated the irradiation conditions and physical characteristics of individual patients. These analyses were based on TFCA patient data obtained in a real clinical setting using contemporary biplane angiography equipment. By presenting empirically based dose evaluations and conversion factors reflecting the clinical environment, it provided data that can sufficiently and quickly convert the patient’s effective dose into actual clinical practice using only the KAP.

## 5. Conclusions

This study quantitatively evaluated the radiation exposure received by patients during diagnostic TFCA and presented the effective dose, organ-absorbed dose, and KAP-based effective dose conversion factors applicable in clinical settings. These efforts allow clinical medical staff to quickly evaluate a patient’s effective dose without undertaking complex computational simulation processes. This shows that the effective dose can be estimated easily and consistently by utilizing the KAP values recorded on the equipment during TFCA examinations, indicating practical applicability for clinical dose monitoring and radiation protection management.

Furthermore, this study quantitatively demonstrated the distribution of organ-absorbed doses, differences in effective doses and radiation-induced risks according to sex, and that TFCA examinations can deliver high doses to radiation-sensitive organs in the head and neck. These findings indicate the necessity of dose management that considers sex and radiation protection standards. In this study, the effective dose, organ-absorbed dose, and KAP-based conversion factors presented serve as data reflecting the radiation dose characteristics of TFCA patients, indicating that efforts must be made for radiation protection and patient safety regarding the inevitable radiation exposure that occurs during interventional procedures in clinical practice.

Furthermore, the procedural framework of this research can be extended to develop conversion factors for other common interventional procedures. Applying these factors according to the procedure type will enable clinicians to easily estimate patient radiation exposure in real-world clinical environments.

Because this is a retrospective, single-center study utilizing specific equipment, the generalizability of our findings to other clinical environments or hardware may be limited. However, this limitation is counterbalanced by the use of real-world patient morphologies and actual clinical cerebral angiography datasets. To ensure procedural consistency and eliminate inter-operator variability, all examinations were performed by a single board-certified neurosurgeon with five years of specialized experience in TFCA procedures.

While the cohort of 30 patients (15 males and 15 females) might initially appear small, it is statistically sufficient according to the Central Limit Theorem (N≥30), which justifies the assumption of normality. To further mitigate the limitations of the sample size and ensure high statistical power, we strictly controlled confounding variables. This was achieved by standardizing the operator, imaging projections, and patient body weight (restricted to the 25th–75th percentiles of the standard Korean physique). Such rigorous control minimized data variance and yielded a robust effect size, as evidenced by the highly significant difference (p<0.001) observed between genders in the independent *t*-test. Ultimately, the high reliability ($R^{2}=0.989$) and low error rate (MAPE = 0.67%) demonstrated through the LOOCV approach confirm that the conversion factors derived from this cohort are both statistically robust and clinically generalizable.

## Figures and Tables

**Table 1:** Descriptive Statistics of KAP and Effective Dose (ICRP 60 and ICRP 103).

	*N*	Minimum	Maximum	Average	SD
KAP (Gy·cm^2^)	30	26.71	55.94	36.23	6.81
ICRP60	30	1.22	2.56	1.70	0.35
ICRP103	30	1.34	2.88	1.90	0.40

SD, standard deviation; KAP, Kerma area product; ICRP, International Commission on Radiological Protection.

**Table 2:** Comparison of KAP and Effective Dose for Male and Female Patients.

	Total	Male	Female
Mean ICRP60 (mSv)	1.70	1.65	1.74
Mean ICRP103 (mSv)	1.90	1.84	1.97
KAP (Gy·cm^2^)	36.23	37.98	34.48

ICRP, International Commission on Radiological Protection; KAP, Kerma area product.

**Table 3:** Absorbed Dose by Organ Based on Monte Carlo Simulation Results.

Tissue	Organ Absorbed Dose (mGy)					
	Total		Female		Male	
	Mean	SD	Mean	SD	Mean	SD
Upper spine	28.66	5.58	27.15	4.39	30.16	6.52
Skull	23.26	4.14	22.09	3.45	24.44	4.68
Scapulae	22.52	5.68	23.77	5.59	21.26	5.88
Clavicle	15.10	2.44	14.71	2.53	15.50	2.47
Upper arm bones	13.48	2.92	14.23	2.69	12.73	3.13
Salivary glands	10.49	2.04	9.92	1.63	11.06	2.36
Middle spine	9.74	2.37	10.34	2.46	9.14	2.28
Ribs	8.77	2.26	9.53	2.31	8.01	2.10
Brain	8.76	1.57	8.31	1.33	9.20	1.76
Thyroid	8.48	1.51	8.08	1.29	8.88	1.69
Skeleton	7.36	1.37	7.37	1.27	7.35	1.56
Extra thoracic airways	6.80	1.25	6.45	1.01	7.15	1.44
Oral mucosa	6.63	1.18	6.33	0.95	6.94	1.37
Lungs	3.65	0.99	4.01	1.00	3.30	0.92
Esophagus	2.84	0.65	2.99	0.66	2.70	0.66
Active bone marrow	2.80	0.57	2.86	0.55	2.73	0.62
Lymph nodes	2.76	0.51	2.71	0.44	2.81	0.59
Skin	2.60	0.48	2.57	0.43	2.62	0.56
Middle arm bones	2.22	0.85	2.73	0.79	1.71	0.61
Thymus	1.66	0.40	1.79	0.38	1.54	0.41
Muscle	1.40	0.28	1.43	0.27	1.37	0.31
Heart	1.31	0.40	1.49	0.40	1.12	0.35
Breasts	1.08	0.35	1.24	0.34	0.93	0.31
Liver	0.37	0.18	0.49	0.17	0.26	0.09
Adrenals	0.37	0.21	0.49	0.22	0.24	0.09
Pancreas	0.35	0.17	0.45	0.17	0.25	0.10
Spleen	0.32	0.17	0.43	0.17	0.22	0.09
Stomach	0.28	0.13	0.36	0.12	0.20	0.07
Gall bladder	0.12	0.07	0.16	0.08	0.08	0.03
Lower spine	0.11	0.06	0.14	0.06	0.07	0.03
Kidneys	0.10	0.05	0.13	0.05	0.07	0.03
Lower arm bones	0.03	0.02	0.04	0.02	0.02	0.01
Upper large intestine	0.02	0.01	0.03	0.01	0.02	0.01
Small intestine	0.02	0.01	0.03	0.01	0.01	0.01
Colon large intestine	0.02	0.01	0.02	0.01	0.01	0.00
Pelvis	0.01	0.01	0.01	0.01	0.01	0.00
Ovaries	0.01	0.01	0.01	0.01	0.00	0.01
Uterus	0.01	0.00	0.01	0.00	0.00	0.00
Lower large intestine	0.00	0.00	0.01	0.00	0.00	0.00
Urinary bladder	0.00	0.00	0.00	0.00	0.00	0.00
Prostate	0.00	0.00	0.00	0.00	0.00	0.00
Upper leg bones	0.00	0.00	0.00	0.00	0.00	0.00
Testicles	0.00	0.00	0.00	0.00	0.00	0.00
Middle leg bones	0.00	0.00	0.00	0.00	0.00	0.00
Lower leg bones	0.00	0.00	0.00	0.00	0.00	0.00
Average dose in total body	3.66	0.67	3.86	0.62	3.45	0.70

SD: Standard deviation.

**Table 4:** Conversion Factors (K) Based on ICRP 60 and ICRP 103.

Conversion Factor (K)	Mean ± SD
K60 (mSv/Gy·cm^2^)	0.0468 ± 0.0054
K103 (mSv/Gy·cm^2^)	0.0525 ± 0.0065

SD, standard deviation.

**Table 5:** Radiation Associated Risk of Exposure Induced Death (REID) and Loss of Life Expectancy (LLE) According to Sex.

	Mean ± SD	Male ± SD	Female ± SD
REID (%)	0.0070 ± 0.0028	0.0053 ± 0.0012	0.0088 ± 0.0028
LLE (Hours)	10.56 ± 5.88	7.5 ± 3.30	13.6 ± 6.40

SD, standard deviation; REID, radiation associated risk of exposure induced death; LLE, loss of life expectancy.

## Data Availability

The data presented in this study are available on request from the corresponding author due to privacy and ethical restrictions.

## Abbreviations

The following abbreviations are used in this manuscript:

TFCA	Transfemoral cerebral angiography
KAP	Kerma area product
DICOM RDSR	Digital imaging and communication in medicine radiation dose structured report
ICRP	International Commission on Radiological Protection
ALARA	As low as reasonably achievable
PACS	Picture archiving and communication system
AP	Anteroposterior
CCA	Cranial caudal angle
K	Conversion factor
E	Effective dose
LLE	Loss of life expectancy
REID	Radiation-associated risk of exposure-induced death

## References

[1] Mettler F.A., Bhargavan M., Faulkner K., Gilley D.B., Gray J.E., Ibbott G.S., Lipoti J.A., Mahesh M., McCrohan J.L., Stabin M.G. (2009). Radiologic and nuclear medicine studies in the United States and worldwide: Frequency, radiation dose, and comparison with other radiation sources—1950–2007. Radiology.

[2] United Nations Scientific Committee on the Effects of Atomic Radiation (2022). Sources, Effects and Risks of Ionizing Radiation: UNSCEAR 2020/2021 Report.

[3] Miller D.L., Vañó E., Bartal G., Balter S., Dixon R., Padovani R., Schueler B., Cardella J.F., De Baère T. (2010). Occupational radiation protection in interventional radiology: A joint guideline of the Cardiovascular and Interventional Radiology Society of Europe and the Society of Interventional Radiology. CardioVasc. Interv. Radiol..

[4] Valentin J. (2008). The 2007 Recommendations of the International Commission on Radiological Protection.

[5] Guide S.S. (2018). Radiation Protection and Safety in Medical Uses of Ionizing Radiation.

[6] Cousins C., Miller D.L., Bernardi G., Rehani M.M., Schofield P., Vañó E., Einstein A.J., Geiger B., Heintz P., Padovani R. (2011). ICRP PUBLICATION 120: Radiological Protection in Cardiology.

[7] Ge B., Wei Y. (2020). Comparison of transfemoral cerebral angiography and transradial cerebral angiography following a shift in practice during four years at a single center in China. Med. Sci. Monit..

[8] Shin D.S., Yeo D.K., Hwang S.C., Park S.Q., Kim B.T. (2013). Protocols and results of resident neurosurgeon’s transfemoral catheter angiography training supervised by neuroendovascular specialists. J. Korean Neurosurg. Soc..

[9] Riabroi K., Khanungwanitkul K., Wattanapongpitak P., Krisanachinda A., Hongsakulet K. (2018). Patient radiation dose in neurointerventional radiologic procedure: A tertiary care experience. Neurointervention.

[10] Ciraj-Bjelac O., Rehani M.M. (2014). Eye dosimetry in interventional radiology and cardiology: Current challenges and practical considerations. Radiat. Prot. Dosim..

[11] Jung K.H., Lee K.Y., Kim H.D., Baek C.H. (2025). Assessment of radiation exposure and effective dose according to imaging conditions in cerebrovascular interventional procedures. New Phys. Sae Mulli.

[12] Korean Standard Body Size Data Center. https://sizekorea.kr/human-meas-search/human-data-search/meas-item.

[13] (2012). Medical Electrical Equipment—Part 2–43: Particular Requirements for the Basic Safety and Essential Performance of X-Ray Equipment for Interventional Procedures.

[14] Feygelman V.M., Huda W., Peters K.R. (1992). Effective dose equivalents to patients undergoing cerebral angiography. Am. J. Neuroradiol..

[15] Yi H.J., Sung J.H., Lee D.H., Kim SWLee S.W. (2017). Analysis of radiation doses and dose reduction strategies during cerebral digital subtraction angiography. World Neurosurg..

[16] Ihn Y.K., Kim B.S., Jeong H.W., Suh S.H., Won Y.D., Lee Y.J., Kim D.J., Jeon P., Ryu C.W., Suh S. (2021). Monitoring radiation doses during diagnostic and therapeutic neurointerventional procedures: Multicenter study for establishment of reference levels. Neurointervention.

[17] Özgür E., Kayaokay D.T., Özgür D.S., Tünayd A., Günaye O., Kesmezacarf F.F., Demirg M., ALMisnedh G., Tekini H.O. (2026). Critical organ dose evaluation during interventional stroke imaging: Implications for radiation safety. Radiography.

[18] Narendran N., Luzhna L., Kovalchuk O. (2019). Sex difference of radiation response in occupational and accidental exposure. Front. Genet..

[19] Safari M.J., Wong J.H.D., Kadir K.A.A., Thorpe N.K., Cutajar D.L., Petasecca M., Lerch M.L.F., Rosenfeld A.B., Ng K.H. (2016). Real-time eye lens dose monitoring during cerebral angiography procedures. Eur. Radiol..

[20] Sanchez R.M., Vano E., Fernández J.M., Moreu M., Lopez-Ibor L. (2014). Brain radiation doses to patients in an interventional neuroradiology laboratory. Am. J. Neuroradiol..

